# Rotating flow assessment of magnetized mixture fluid suspended with hybrid nanoparticles and chemical reactions of species

**DOI:** 10.1038/s41598-021-90519-6

**Published:** 2021-05-28

**Authors:** Noor Saeed Khan, Qayyum Shah, Arif Sohail, Zafar Ullah, Attapol Kaewkhao, Poom Kumam, Seema Zubair, Naeem Ullah, Phatiphat Thounthong

**Affiliations:** 1 KMUTTFixed Point Research Laboratory, Room SCL 802 Fixed Point Laboratory, Science Laboratory Building, Department of Mathematics, Faculty of Science, King Mongkut’s University of Technology Thonburi (KMUTT), Bangkok, 10140 Thailand; 2 Center of Excellence in Theoretical and Computational Science (TaCS-CoE), Science Laboratory Building, Faculty of Science, King Mongkut’s University of Technology Thonburi (KMUTT), 126 Pracha-Uthit Road, Bang Mod, Bangkok, Bangkok, 10140 Thailand; 3 Department of Medical Research, China Medical University Hospital, China Medical University, Taichung, 40402 Taiwan; 4 Division of Science and Technology, Department of Mathematics, University of Education, Lahore, 54000 Pakistan; 5 Department of Basic Sciences and Islamiyat, University of Engineering and Technology, Peshawar, Khyber Pakhtunkhwa 2500 Pakistan; 6 Research Center in Mathematics and Applied Mathematics, Faculty of Science, Chiang Mai University, Chiang Mai, 50200 Thailand; 7 Renewable Energy Research Centre, Department of Teacher Training in Electrical Engineering, Faculty of Technical Education, King Mongkut’s University of Technology North Bangkok, 1518 Pracharat 1 Road, Wongsawang, Bangsue, Bangkok, 10800 Thailand; 8 Department of Mathematics, Statistics and Comuter Science, The University of Agriculture, Peshawar, Khyber Pakhtunkhwa 25130 Pakistan; 9 Department of Mathematics, Islamia College University, Peshawar, Khyber Pakhtunkhwa 25000 Pakistan

**Keywords:** Energy science and technology, Engineering, Materials science, Mathematics and computing, Nanoscience and technology, Physics

## Abstract

The current study characterizes the effects of Hall current, Arrhenius activation energy and binary chemical reaction on the rotating flow of hybrid nanofluid in two double disks. By the use of suitable similarity transformations, the system of partial differential equations and boundary conditions for hybrid nanofluid are transformed to ordinary differential equations which are solved through optimal homotopy analysis method. The intensified magnetic field and hybrid nanofluid performances are represented in three dimensional model with flow, heat and mass transfer. Radial velocity decreases and tangential velocity increases with the Hall parameter. Temperature rises with high values of rotation parameter while it decreases with the Prandtl number. Nanoparticles concentration enhances with the increments in Arrhenius activation energy parameter and stretching parameter due to lower disk. There exists a close and favorable harmony in the results of present and published work.

## Introduction

The analysis of chemical reaction has numerious applications such as food processing, polymer production, synthesis and oxidation of materials, contamination, biochemical engineering, metallurgy and plastic expulsion, chemical processing types of equipments, evaporations, manufacturing of ceramics, energy transfer in a drizzly cooling tower, etc. Ali et al.^1^ worked on the finite element method to prepare the code for the chemical reactions, heat source, magnetic field, thermal radiation, activation energy, and convective boundary conditions to present the parametric computations for faster stretch and slowly stretch to the surface of the wedge. Hayat et al.^2^ devoted to the convection and mass transfer flow of an electrically conducting viscous fluid on a curved surface with chemical reactions. Bibi and Xu^3^ discussed the characteristics of homogeneous–heterogeneous chemical reactions in peristaltic flow of Carreau magneto hybrid nanofluid with copper and silver nanoparticles in a symmetric channel with velocity slip condition, thermal radiation and entropy generation signifying that hybrid nanofluid has better thermal conductivity compared to the conventional nanofluid. Sambath et al.^4^ presented the thermal radiation, chemical reaction and heat source/sink for laminar natural convective MHD flow of viscous incompresible gray absorbing and emitting, but non-scattering fluid past a vertical cone considering the variable wall temperature and concentration by using implicit finite difference method of Crank–Nicolson having speedy convergence and stability. Sohail et al.^5^ reported the chemical reaction with entropy generation and variable properties of magnetic field, thermal conductivity, diffusion coefficient for the Couple stress model. They compared their results in limiting case which provides an excellent agreement. Some other studies related to chemical reactions are in the references^6–11^.

Magnetohydrodynamics analyzes the dynamical behavior of electrically conducting fluids such as plasma, liquid metals and electrolytes or salt water. Its applications can be seen in hyperthermia, magnetic cell separation, treatment of some arterial diseases, drug delivery. Lund et al.^12^ used the similarity variables of transformations to study the steady, two-dimensional, stagnation point and magnetohydrodynamic flow on an exponentially vertical stretching/shrinking surface with convective boundary conditions.They obtained two ranges of solutions in the specific ranges of the physical parameters where three solutions corrospond to the opposing flow. Siddiqui et al.^13^ presented the numerical solution of magnetohydrodynamic mixed convection within a lid steered square geometry having micropolar fluid. They used the finite element method in addition to Galerkin weighted residual to get the outcomes. Islam et al.^14^ analyzed the micropolar ferrofluid past a stretching sheet with the effect of manetohydrodynamics using the convective and slip conditions employing homotopy analysis method. Beg et al.^15^ worked on the steady, incompressible, laminar Newtonian magnetohydrodynamic slip flow with heat transfer in spinning porous disk with strong injection, thermal radiation and fluid thermophysical properties. Agrawal et al.^16^ examined the applied magnetic field effect on incompressible, free convective boundary layer flow past a stretching porous space with temperature dependent viscosity and heat source/sink. They used Lie group similarity transformation to achieve the symmetric graphs of the problem. El-Kabeir^17^ applied the group theoretic method for computing the problem of magnetohydrodynamic heat and mass transfer non-Darcy flow in an impermeable horizontal cylinder. The other magnetohydrodynamics studies can be seen in the references^18–22^.

Hall current effect which is generated due to the applied magnetic field of high intensity has an important role in engineering such as geophysics, cosmological fluid dynamics, Hall accelerator, etc. while in medical sciences, it has applications like cardiac MRI, ECG, etc. Khan et al.^23^ analyzed the Hall current effect on the hybrid nanofluid flow past an unsteady rotating disk. Singh et al.^24^ presented the exact solution in closed form for the Hall effect on steady hydromagnetic mixed convective generalised Couette flow between two infinite parallel plates of arbitrary electrical conductivities and finite thickness filled with porous medium in the presence of a uniform transverse magnetic field in a rotating system. Abdel-Wahed and Akl^25^ investigated the Hall current effect on the MHD flow of nanofluid with variable properties due to a rotating disk with viscous dissipation and nonlinear thermal radiation using the solution of optimal homotopy asymptotic metod (OHAM). Gosh et al.^26^ derived the closed form solution for the steady magnetohydrodynamic viscous flow in a parallel plate channel system with perfectly conducting walls in a rotating frame of reference with Hall current, heat transfer and a transverse magnetic field. They found that boundary layers increase close to the channel walls for the high values of rotation parameter and for slowly rotating system, Hall current parameter decreases primary mass flow rate. By using Saffman’s proposed model for the suspension of fine dust particles, Bilal and Ramzan^27^ discussed the unsteady two-dimensional flow of mixed convection and nonlinear thermal radiation in water based carbon nanotubes for Hall current effect. Ahmad et al.^28^ investigated Hall current effect, Brownian motion, thermophoresis, entropy generation, thermal radiation, Joule heating and heat source/sink for the second-grade nanofluid flow with Cattaneo–Christov heat flux model. Explanation about Hall current effect can be read in the references^29–31^.

Heat transfer is the principal target for researchers due to its applications in evaporators, condensers, air conditioning systems, power generations. Adding one or more types of nanoparticles to the base fluid is one of the methods to promote the rate of heat transfer. Ali et al.^32^ generated the closed form solution of the laminar and unsteady Couple stress nanofluid flow with based fluid and nanoparticles as engin oil and Molybdenum disulphide nanoparticles respectively. They noticed that rate of heat transfer of engin oil is enhanced up to 12.38$\%$ by the inclusion of Molybdenum disulphide nanoparticles in the base fluid. Imtiaz et al.^33^ investigated the blood flow with gold nanomaterials in a cylidrical tube under the oscillating pressure gradient and magnetic field using Caputo Fabrizio and Atangana–Baleanue derivatives approaches. Kotnurkar^34^ presented the bioconvective peristaltic flow of a third-grade nanofluid flow with Cu-blood nanoparticles and gyrotactic microorganisms. They proved that the thermophoresis and Brownian motion parameters increase the heat transfer and Prandtl number has decreasing effect. Hayat et al.^35^ addressed the Darcy–Forchheimer flow of viscous nanofluid saturating the porous medium. Their results show that the local Nusselt and Sherwood numbers are diminished due to high values of local porosity parameter. Influencial studies about nanofluids can be reffered to the references^36–42^.

Maraj et al.^43^ carried out a study about a comprehensive shape factor investigations of $\hbox {MoS}_{2}$-$\hbox {SiO}_{2}$ water based hybrid nanofluid in a semi vertical inverted porous cone. They used the shooting algorithm to find that motion decreases more for $\hbox {SiO}_{2}/\hbox {H}_{2}\hbox {O}$ nanofluid as compared to hybrid nanofluid and heat transfer is maximum achieved with increasing Eckert number, volumetric fractions. Salehi et al.^44^ reported the water and glycol based hybrid nanoparticles problem solved through Akbari–Ganji’ method. They showed that velocity is decreasd and temperature is increased by increasing the squeez number. Shah et al.^45^ used control volume finite element method to solve numerically the problem of non-Darcy MHD hybrid nanofluid in a porous tank with entropy generation. Khan et al.^46^ investigated the entropy optimization in MHD propylene glycol based hybrid nanofluid via Newton built-in shooting method by finding that molybdenum disulfide has better efficiency compared to silicon dioxide. Wahid et al.^47^ discussed the hybrid nanofluid slip flow of water based alumina and copper nanoparticles in the presence of heat generation past an exponenetially stretching/shrinking porous sheet. Their obtained solution through bvp4c in Matlab software shows that the rise in volume fraction of copper nanoparticles increases the skin friction coefficient and Nusselt number. Muhammad et al.^48^ analyzed the flow of gasoline based hybrid nanofluid containing single walled carbon nanotubes and multi walled carbon nanotubes on a curved stretched surface. Using shooting method, they presented the relative analysis of base fluid, single walled carbon nanotubes and hybrid nanofluid. Similar studies on hybrid nanofluids are exist in the references^49,51^.

The present paper reflects on the Hall current effect, Arrhenius activation energy and binary chemical reactions on three dimensional flow of hybrid nanofluid. Optimal homotopy analysis method^52,53^ is used to generate the solution of the non-dimensional equations. Graphically interpretations are made with the help of different embedded parameters.

## Methods

### Basic equations

**Figure 1:**
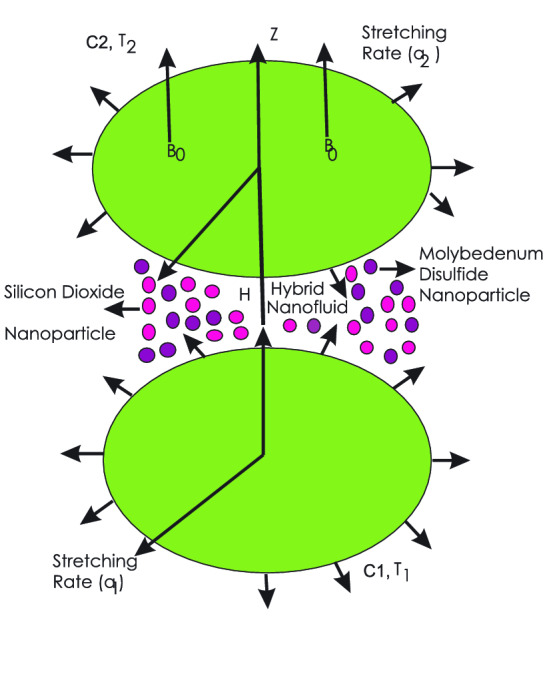
Geometry of the problem.

The axisymmetric motion of magnetohydrodynamic three dimensional, time independent and an incompressible nanofluid between two parallel infinite disks is considered. The lower disk is supposed to lie at $\textit{z} = 0$. The distance between upper and lower disks is *H*. The lower and upper disks have the angular velocities $\Omega _{1}$ and $\Omega _{2}$ respectively in the rotation of axial direction. The stretching rates, temperatures and concentrations on the lower and upper disks are ($a_{1}$, $T_{1}$, $C_{1}$) and ($a_{2}$, $T_{2}$, $C_{2}$) respectively. An intensified magnetic field of strength $B_{0}$ is applied in the *z*-direction (see Fig. 1). The base fluid is water in which silicon dioxide and molybedenum disulfide nanoparticles are added.

Under the application of cylindrical coordinates (*r*, $\vartheta$, *z*), the governing equations of the hybrid nanofluid are as in^23–28,52^

1 $$\begin{aligned}&\frac{\partial u}{\partial r} + \frac{u}{r} +\frac{\partial w}{\partial z} = 0, \end{aligned}$$

2 $$\rho _{hnf}\begin{pmatrix} u \frac{\partial u}{\partial r} +w \frac{\partial u}{\partial z} -\frac{v^{2}}{r} \end{pmatrix} =-\frac{\partial P}{\partial r} + \mu _{hnf} \begin{pmatrix} \frac{1}{r}\frac{\partial u}{\partial r} - \frac{u}{r^{2}} + \frac{\partial ^{2} u}{\partial r^{2}} + \frac{\partial ^{2} u}{\partial z^{2}} \end{pmatrix} -\frac{\sigma _{hnf} B^{2} _{0}\ (u - mv)}{1 + m^{2}}, $$

3 $$\rho _{hnf}\begin{pmatrix} u \frac{\partial v}{\partial r} + w \frac{\partial v}{\partial z} + \frac{uv}{r} \end{pmatrix} =\mu _{hnf}\begin{pmatrix} \frac{1}{r}\frac{\partial v}{\partial r} - \frac{v}{r^{2}} +\frac{\partial ^{2} v}{\partial r^{2}} + \frac{\partial ^{2} v}{\partial z^{2}} \end{pmatrix} -\frac{\sigma _{hnf} B^{2} _{0}\ (v + mu)}{1 + m^{2}}, $$

4 $$\begin{aligned}\rho _{hnf}\begin{pmatrix} u \frac{\partial w}{\partial r}+w \frac{\partial w}{\partial z} \end{pmatrix} =-\frac{\partial P}{\partial z} + \mu _{hnf}\begin{pmatrix} \frac{1}{r}\frac{\partial w}{\partial r} + \frac{\partial ^{2} w}{\partial r^{2}} +\frac{\partial ^{2} w}{\partial z^{2}} \end{pmatrix}, \end{aligned}$$

5 $$(\rho c_{p})_{hnf} \begin{pmatrix}u \frac{\partial T}{\partial r} +w \frac{\partial T}{\partial z}  \end{pmatrix}= \begin{pmatrix}k_{hnf} + \frac{16T^{3}_{1}\sigma _{1}}{3k_{1}}   \frac{1}{r}\end{pmatrix}\begin{pmatrix}\frac{\partial T}{\partial r} + \frac{\partial ^{2} T}{\partial r^{2}} +\frac{\partial ^{2} T}{\partial z^{2}}\end{pmatrix} +\sigma _{hnf} B^{2}_{0}(u^{2} + v^{2}), $$

6 $$u \frac{\partial C}{\partial r} + w \frac{\partial C}{\partial z} =D_{B} \begin{pmatrix} \frac{1}{r}\frac{\partial C}{\partial r} +\frac{\partial ^{2} C}{\partial r^{2}} +\frac{\partial ^{2} C}{\partial z^{2}} \end{pmatrix} -k_{r}^{2}(\textit{C} - C_{2})\begin{bmatrix} \frac{T}{T_{2}} \end{bmatrix}^{m_{1}} \exp \begin{bmatrix} \frac{E_{a}}{\sigma _{1} T} \end{bmatrix}. $$

The boundary conditions are used as

7 $$\begin{aligned}&u = \textit{r}a_{1},\quad v = \textit{r}\Omega _{1}, \quad w = 0, \quad T = T_{1}, \quad C = C_{1}, \quad at \quad z = 0, \end{aligned}$$

8 $$\begin{aligned}&u = \textit{r}a_{2},\quad v = \textit{r}\Omega _{2}, \quad w = 0, \quad T = T_{2}, \quad \textit{C} = C_{2}, \quad at \quad z = \textit{H}, \end{aligned}$$

where the components of velocity are *u*(*r*, $$\vartheta $$, *z*), *v*(*r*, $$\vartheta $$, *z*) and *w*(*r*, $$\vartheta $$, *z*), *P* manifests the pressure, the magnetic induction is $$B = (0, 0, B_{0})$$, *m* is the Hall parameter^23–28,52^, $$k_{1} $$ is mean absorption coefficient, $$ \sigma _{1}= 8.61 \times 10^{-5}\hbox {eV}/\hbox {K}$$ is the Stefan–Boltzmann constant and *T* is the fluid temperature, $$m_{1}$$ is the fitted rate constant such that ($$-1< m_{1}< 1$$), $$E_{a}$$ is the activation energy in which *a* is the positive dimensional constant and $$k_{r}^{2}(\textit{C} - C_{2})\begin{bmatrix} \frac{T}{T_{2}} \end{bmatrix}^{m_{1}}\exp \begin{bmatrix} \frac{E_{a}}{\sigma _{1} T} \end{bmatrix}$$ is the modified Arrhenius term. For the hybrid nanofluid, the important quantities are $$\rho _{hnf}$$ (density), $$\mu _{hnf}$$ (dynamic viscosity), $$\sigma _{hnf}$$ (electrical conductivity), $$(c _{p})_{hnf}$$ (heat capacity) and $$k _{hnf}$$ (thermal conductivity). The subscript “hnf” shows the hybrid nanofluid. The subscript “*nf*” is used for the nanofluid. The subscript “f” is used for the base fluid. $$\phi _{1}$$ is the first nanoparticle volumetric fraction while $$\phi _{2}$$ is the second nanoparticle volume fraction which are expressed as

9 $$\begin{aligned}&\rho _{s} = \frac{(m_{2} \times \rho _{1}) + (m_{3} \times \rho _{2})}{m_{2} + m_{3}}, \end{aligned}$$

10 $$\begin{aligned}&(c_{P})_{s} = \frac{(m_{2} \times (c_{P})_{1}) +(m_{3} \times (c_{P})_{2})}{m_{2} + m_{3}}, \end{aligned}$$

11 $$\begin{aligned}&\phi _{1} = \frac{\frac{m_{2}}{\rho _{1}}}{\frac{m_{f}}{\rho _{f}} +\frac{m_{2}}{\rho _{1}} + \frac{m_{3}}{\rho _{2}}}, \end{aligned}$$

12 $$\begin{aligned}&\phi _{2} = \frac{\frac{m_{3}}{\rho _{2}}}{\frac{m_{f}}{\rho _{f}} +\frac{m_{2}}{\rho _{1}} + \frac{m_{3}}{\rho _{2}}}, \end{aligned}$$

13 $$\begin{aligned}&\phi = \phi _{1} + \phi _{2}, \end{aligned}$$

where $m_{f}$, $m_{2}$ and $m_{3}$ are respectively the mass of the base fluid, masses of the initial and second nanoparticles. $\phi$ stands for total nanoparticles concentration of silicon dioxide and molybedenum disulfide nanoparticles.

The thermophysical properties of $\hbox {H}_{2}\hbox {O}$ and nanoparticles are given in Table 1^46^. The important and relevant information is given in Table 2^50^ in which $\phi _{s}$ is used for the solid particle concentration.

**Table 1:** Thermophysical properties of water and nanoparticles^46^.

Properties	Water ($\hbox {H}_{2}\hbox {O}$)	Silicon dioxide ($\hbox {SiO}_{2}$)	Molybedenum disulfide ($\hbox {MoS}_{2}$)
$\rho (\hbox {kg}/\hbox {m}^{3})$	$\rho _{f} = 997.1$	$\rho _{s_{1}} = 2650$	$\rho _{s_{2}} = 5060$
$\hbox {c} _{P}$(J/kg K)	$(\hbox {c} _{P})_{f} = 4179$	$(\hbox {c} _{P})_{s_{1}} = 730.0$	$(\hbox {c} _{P})_{s_{2}} = 397.746$
*k*(W/m K)	$k_{f} = 0.613$	$k_{s_{1}} = 1.5$	$k_{s_{2}} = 34.5$
$\sigma (\Omega \,\hbox {m})^{-1}$	$\sigma _{f} = 0.05$	$\sigma _{s_{1}} =1.0\times 10^{-18}$	$\sigma _{s_{2}} = 2.09\times 10^{4}$
$\beta$(1/K)	$\beta _{f} = 21.0$	$\beta _{s_{1}} = 42.7$	$\beta _{s_{2}} = 2.8424\times 10^{-5}$

**Table 2:** Mathematical expression of thermophysical properties^50^.

Properties	$\hbox {SiO}_{2}/\hbox {H}_{2}\hbox {O}$
Density ($\rho$)	$\rho _{nf} = (1 - \phi _{1})\rho _{f}+\phi _{1}\rho _{s}$
Heat capacity ($\rho \hbox {c} _{P}$)	$(\rho \hbox {c}_{P})_{nf} =(1 -\phi _{1})(\rho \hbox {c}_{P})_{f} +\phi _{1}(\rho \hbox {c}_{P})_{s}$
Dynamic viscosity ($\mu$)	$\frac{\mu _{nf}}{\mu _{f}}=\frac{1}{(1-\phi _{1})^{2.5}}$
Thermal conductivity (*k*)	$\frac{k_{nf}}{k_{f}}=\frac{k_{s_{1}} + (n_{1} - 1) k_{f} - (n_{1} - 1)(k_{f} - k_{s_{1}})\phi _{1}}{k_{s_{1}} + (n_{1} - 1)k_{f} + (k_{f} - k_{s})\phi _{1}}$
Electrical conductivity ($\sigma$)	$\frac{\sigma _{nf}}{\sigma _{f}} = 1 + \frac{3(\sigma - 1)\phi _{1}}{(\sigma + 2) - (\sigma - 1)\phi _{1}}$, where $\sigma = \frac{\sigma _{s}}{\sigma _{f}}$
Properties	Hybrid nanofluid ($\hbox {MoS}_{2}$–$\hbox {SiO}_{2}/\hbox {H}_{2}\hbox {O}$)
Density ($\rho$)	$\rho _{hnf} = (1 - (\phi _{1} + \phi _{2}))\rho _{f}+ \phi _{1}\rho _{s_{1}}+\phi _{2}\rho _{s_{2}}$
Heat capacity ($\rho \hbox {c} _{P}$)	$(\rho \hbox {c}_{P})_{nf}= (1-(\phi _{1}+\phi _{2})) (\rho \hbox {c}_{P})_{f}+\phi _{1}(\rho \hbox {c}_{P})_{s_{1}} +\phi _{2}(\rho \hbox {c}_{P})_{s_{2}}$
Dynamic viscosity ($\mu$)	$$\frac{\mu _{hnf}}{\mu _{f}} = \frac{1}{\begin{bmatrix}1 - (\phi _{1} + \phi _{2})\end{bmatrix}^{2.5}}$$
Thermal conductivity (*k*)	$\frac{k_{hnf}}{k_{f}} = \frac{k_{s_{2}} + (n_{2} - 1)k_{nf} - (n_{2} - 1)(k_{nf} - k_{s_{2}})\phi _{2}}{k_{s_{2}} + (n_{2} - 1)k_{nf} + (k_{nf} - k_{s_{2}})\phi _{2}}\times \frac{k_{s_{1}} + (n_{1} - 1)k_{f} - (n_{1} - 1)(k_{f} - k_{s_{1}})\phi _{1}}{k_{s_{1}} + (n_{1} - 1)k_{f} + (k_{f} - k_{s_{1}})\phi _{1}}\times \hbox {k}_{f}$
Electrical conductivity ($\sigma _{hnf}$)	$$\frac{\sigma _{hnf}}{\sigma _{f}}= 1 +\frac{3\begin{bmatrix}\frac{\sigma _{1}\phi _{1} + \sigma _{2}\phi _{2}}{\sigma _{f}} -(\phi _{1} + \phi _{2})\end{bmatrix}}{2 + \begin{bmatrix}\frac{\sigma _{1}\phi _{1} +\sigma _{2}\phi _{2}}{(\phi _{1} + \phi _{2})\sigma _{f}}\end{bmatrix} -\begin{bmatrix}\frac{\sigma _{1}\phi _{1} + \sigma _{2}\phi _{2}}{\sigma _{f}}-(\phi _{1} + \phi _{2})\end{bmatrix}}$$

Following transformations are used

14 $$u = \textit{r}\Omega _{1}f^{\prime }(\zeta ),\quad \textit{v} = \textit{r}\Omega _{1} \textit{g}(\zeta ),\quad \textit{w} = - 2\textit{H}\Omega _{1}{} \textit{f}(\zeta ), \quad \theta (\zeta ) = \frac{T-T_{2}}{T_{1}-T_{2}},\quad \varphi (\zeta ) =\frac{C-C_{2}}{C_{1}-C_{2}},\nonumber \textit{P} = \rho _{f}\Omega _{1}\nu _{f}\begin{pmatrix} \textit{P}(\zeta ) + \frac{r^{2}\epsilon }{2H^{2}} \end{pmatrix}, \quad \zeta = \frac{z}{H}, $$

where $\nu _{f}=\frac{\mu _{f}}{\rho _{f}}$ is the kinematic viscosity and $\epsilon$ is the pressure parameter.

Using the quantities from Eq. (14) and the data of Tables 1 and 2, Eqs. (2–8) provide the following seven Eqs. (15–21).

15 $$B_{1}f^{\prime \prime \prime } + \textit{Re}\begin{bmatrix} 2f {} f ^{\prime \prime } -{f^{\prime }}^{2} + g ^{2} +B_{2}\dfrac{M(f^{\prime } - mg)}{1 + m^{2}} \end{bmatrix} - \epsilon = 0, $$

16 $$B_{1}g^{\prime \prime } + \textit{Re}\begin{bmatrix} 2f {} g ^{\prime } - B_{2} \dfrac{M(mf^{\prime } + g)}{1 + m^{2}} \end{bmatrix} = 0, $$

17 $$\begin{aligned}&P^{\prime } = - 4\textit{Re}{} f {} f ^{\prime } -f ^{\prime \prime }, \end{aligned}$$

18 $$B_{3}\frac{k_{hnf}}{k_{f}}\theta ^{\prime \prime } + \frac{1}{\textit{Rd}} Pr {} \textit{Re}\begin{bmatrix} 2f \theta ^{\prime } + M {} \textit{Ec}B_{4}\begin{pmatrix} g^{2} + (f^{\prime })^{2} \end{pmatrix} \end{bmatrix} = 0,$$

19 $$\varphi ^{\prime \prime } + Re {} \textit{Sc}{} f \phi ^{\prime } +\gamma _{1}(\gamma _{2} + 1)^{m_{1}}{} \textit{Sc}\varphi \exp \begin{bmatrix} \frac{-E}{\gamma _{2}\theta + 1} \end{bmatrix} = 0, = 0, $$

20 $$\begin{aligned}&f = 0, \quad f^{\prime } = k_{2}, \quad g = 1, \quad \theta = 1, \quad \varphi = 1,\quad \textit{P} = 0\quad at \quad \zeta = 0, \end{aligned}$$

21 $$\begin{aligned}&f = 0, \quad f^{\prime } = k_{3}, \quad g = \Omega , \quad \theta = 0, \quad \varphi = 0,\quad at \quad \zeta = 1, \end{aligned}$$

where the notation ($^{\prime }$) is used for differentiation with respect to $\zeta$. $$B_{1} = \begin{bmatrix} 1 - \frac{\frac{m_{1}}{\rho _{1}}}{\frac{m_{1}}{\rho _{1}} +\frac{m_{2}}{\rho _{2}} + \frac{m_{f}}{\rho _{f}}} \end{bmatrix}^{-2.5}\times \begin{bmatrix} 1 - \frac{\frac{m_{1}}{\rho _{1}}}{\frac{m_{1}}{\rho _{1}} +\frac{m_{2}}{\rho _{2}} + \frac{m_{f}}{\rho _{f}}} +\frac{\frac{m_{1}}{\rho _{1}}}{\frac{m_{1}}{\rho _{1}} +\frac{m_{2}}{\rho _{2}} + \frac{m_{f}}{\rho _{f}}}\frac{\rho _{s}}{\rho _{f}} \end{bmatrix}^{-1}$$, $$B_{2} = 1 + \frac{3\begin{bmatrix} \frac{\sigma _{1}\phi _{1} + \sigma _{2}\phi _{2}}{\sigma _{f}} - (\phi _{1} + \phi _{2})\end{bmatrix}}{2 + \begin{bmatrix} \frac{\sigma _{1}\phi _{1} + \sigma _{2}\phi _{2}}{(\phi _{1} + \phi _{2})\sigma _{f}} \end{bmatrix} - \begin{bmatrix}\frac{\sigma _{1}\phi _{1} + \sigma _{2}\phi _{2}}{\sigma _{f}} - (\phi _{1} + \phi _{2})\end{bmatrix}}$$,

$$B_{3} = \frac{(\rho c_{P})_{f}}{\begin{bmatrix}\begin{bmatrix} 1 - \frac{\frac{m_{1}}{\rho _{1}}}{\frac{m_{1}}{\rho _{1}} + \frac{m_{2}}{\rho _{2}} + \frac{m_{f}}{\rho _{f}}} \end{bmatrix} \rho _{f} + \begin{bmatrix} 1 - \frac{\frac{m_{1}}{\rho _{1}}}{\frac{m_{1}}{\rho _{1}} + \frac{m_{2}}{\rho _{2}} + \frac{m_{f}}{\rho _{f}}} \end{bmatrix}\rho _{s}\end{bmatrix} \times \begin{bmatrix}\begin{bmatrix} 1 - \frac{\frac{m_{1}}{\rho _{1}}}{\frac{m_{1}}{\rho _{1}} + \frac{m_{2}}{\rho _{2}} + \frac{m_{f}}{\rho _{f}}} \end{bmatrix}(c_{P})_{f} + \begin{bmatrix} 1 - \frac{\frac{m_{1}}{\rho _{1}}}{\frac{m_{1}}{\rho _{1}} +\frac{m_{2}}{\rho _{2}} + \frac{m_{f}}{\rho _{f}}} \end{bmatrix}(c_{P})_{s}\end{bmatrix}}$$,

$B _{4} = \frac{\sigma _{hnf}}{\rho _{hnf}}$. The other non-dimensional parameters are $\Omega =\frac{\Omega _{2}}{\Omega _{1}}$, $\textit{M} =\frac{\sigma _{f} B^{2}_{0}}{\rho _{f}\Omega _{1}}$, $Rd =\frac{16\sigma _{1}T^{3}_{1}}{3k_{f}k_{0}}$, $\textit{Re} =\frac{\Omega _{1}H^{2}}{\nu _{f}}$, $Pr =\frac{(\rho _{c_{P}})_{hnf}\nu _{f}}{k_{f}}$, $Ec =\frac{r^{2}\Omega ^{2}_{1}}{c_{P}(T_{1} - T_{2})}$, $Sc =\frac{\nu _{f}}{D_{B}}$, $k_{2} =\frac{a_{1}}{\Omega _{1}}$ and $k_{3} = \frac{a_{2}}{\Omega _{1}}$ which are known as rotation, magnet field and thermal radiation parameters, including Reynolds, Prandtl, Eckert and Schmidt numbers and in addition to stretching parameters at lower and upper disk respectively. $\gamma _{1} =\frac{k_{r}^{2}H^{2}}{\nu _{f}}$, $\gamma _{2}=\frac{T_{1} -T_{2}}{T_{2}}$ and $\textit{E} = \frac{E_{a}}{\sigma _{1} T_{2}}$ are the chemical reaction, temperature difference and non-dimensional activation energy parameters respectively.

On account of simplification, diffrentiating Eq. (15) with respect to $\zeta$ presents

22 $$\begin{aligned} B_{1}f^{\prime \prime \prime \prime } + \textit{Re}\begin{bmatrix} 2f {} f ^{\prime \prime \prime } + 2g {} g ^{\prime } +B_{2}\dfrac{M(f^{\prime \prime } - mg^{\prime })}{1 + m^{2}} \end{bmatrix} = 0. \end{aligned}$$

Similarly integration of Eq. (17) with respect to $\zeta$ by using the limit 0 to $\zeta$ provides

23 $$\begin{aligned} P = - 2\begin{bmatrix} \textit{Re}(f )^{2} + \begin{pmatrix} f ^{\prime } - f ^{\prime }(0) \end{pmatrix}\end{bmatrix}. \end{aligned}$$

## Analytical solution

OHAM^52^ is used to solve the non-dimensional Eqs. (16, 18–22). The procedure is followed as choosing the initial guesses and required linear operators for velocities, temperature and concentration profiles as

24 $$\begin{aligned}&f_{0}(\zeta ) = \zeta ^{3}(k_{2} + k_{3}) - \zeta ^{2}(2k_{2} + k_{3}) + \zeta k_{2},\quad g_{0}(\zeta ) = \zeta \Omega + 1 - \zeta , \quad \theta _{0}(\zeta ) = - \zeta + 1,\nonumber \\&\varphi _{0}(\zeta ) = - \zeta + 1, \end{aligned}$$

25 $$\begin{aligned}&\varphi ^{\prime \prime } = {{\varvec{L}}}_{\varphi }, \quad f^{\prime \prime \prime \prime } = {{\varvec{L}}}_{f }, \quad g^{\prime \prime } = {{\varvec{L}}}_{g},\quad \theta ^{\prime \prime } ={{\varvec{L}}}_{\theta }, \end{aligned}$$

characterizing

26 $$\begin{aligned} {{\varvec{L}}}_{f}\begin{bmatrix} E_{1} + E_{2}\zeta + E_{3}\zeta ^{2} + E_{4}\zeta ^{3} \end{bmatrix} = 0, \quad {{\varvec{L}}}_{g}\begin{bmatrix} E_{5} + E_{6}\zeta \end{bmatrix} = 0, \quad {{\varvec{L}}}_{\theta }\begin{bmatrix} E_{7} + E_{8}\zeta \end{bmatrix} = 0, \quad {{\varvec{L}}}_{\varphi }\begin{bmatrix} E_{9} + E_{10}\zeta \end{bmatrix} = 0, \end{aligned}$$

where $E_{i}$($i = 1-10$) are the arbitrary constants.

### Validation of the current work

Solution accuracy is validated by comparing the solution with the published work. Order of approximation of the present work in Table 3 shows the nice agreement with the published literature^11^.

**Table 3:** Comparison of the current work.

Order of approximation	$\hbox {f}^{\prime \prime }$(0)^11^	$\hbox {f}^{\prime \prime }$(0) (Present)	$\hbox {g}^{\prime }$(0)^11^	$\hbox {g}^{\prime }$(0) (Present)
1	1.59936137	1.59936125	0.20487518	0.20487515
2	1.599360685	1.599360681	0.204875662	0.204875661
3	1.59936095	1.59936095	0.204875662	0.204875662
15	1.59936095	1.59936095	0.204875662	0.204875662

## Results and discussion

Results and discussion provide the analysis of the system through the impacts of all relevant representatives. The non-dimensional Eqs. (16, 18–22) are analytically computed through OHAM. The effects of different parameters on the flow profiles, heat and mass transfer with chemical reaction are shown in the relevant graphs. The physical engineering of the problem is shown through Fig. 1.

### Radial and tangential velocity profiles

**Figure 2:**
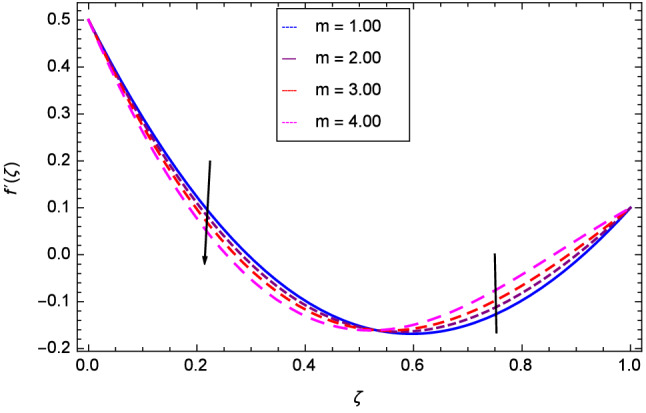
Analysis through Hall parameter *m* and radial velocity $f^{\prime }$($\zeta$).

**Figure 3:**
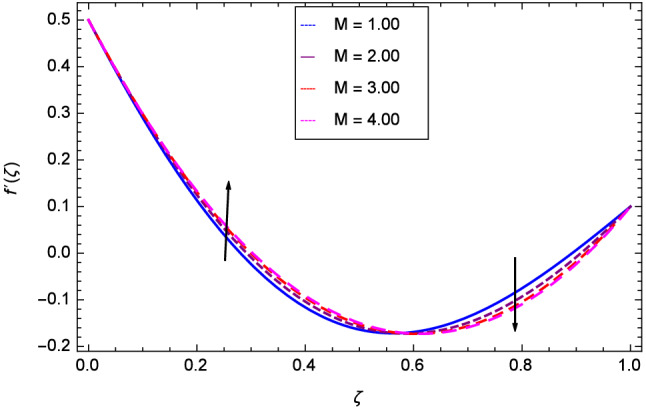
Analysis through magnetic field parameter *M* and radial velocity $f^{\prime }$($\zeta$).

**Figure 4:**
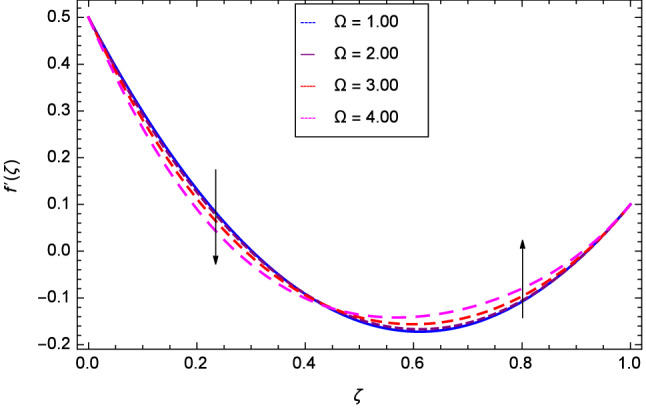
Analysis through rotation parameter $\Omega$ and radial velocity $f^{\prime }$($\zeta$).

**Figure 5:**
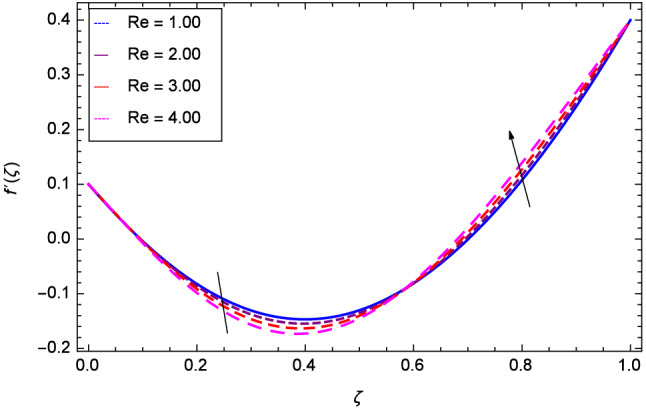
Analysis through Reynolds number *Re* and radial velocity $f^{\prime }$($\zeta$).

**Figure 6:**
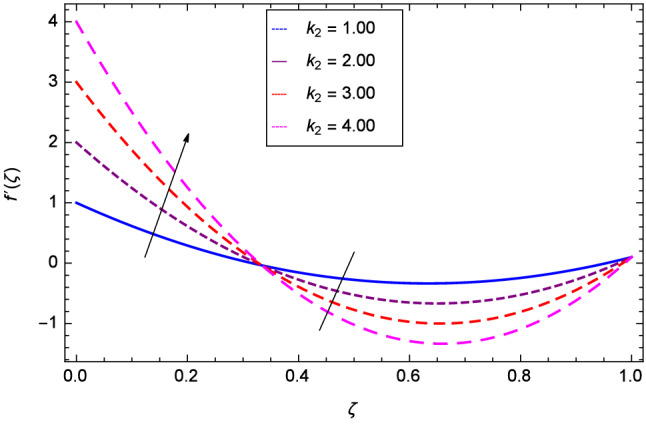
Analysis through lower disk stretching parameter $k_{2}$ and radial velocity $f^{\prime }$($\zeta$).

**Figure 7:**
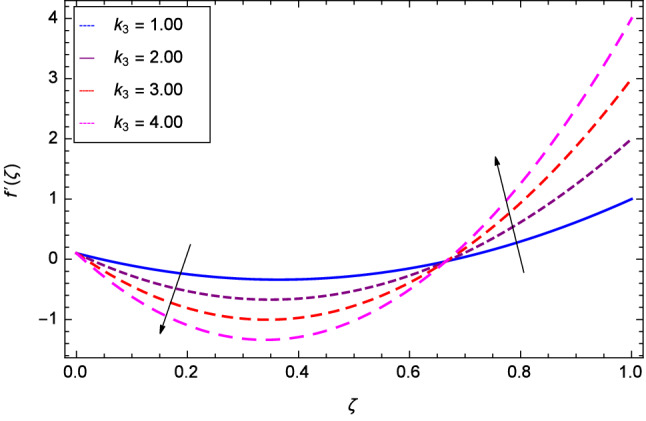
Analysis through upper disk stretching parameter $k_{3}$ and radial velocity $f^{\prime }$($\zeta$).

Hall current effect is generated due to the spiraling of suspension particles about the magnetic lines of force and its direction is mutually perpendicular to the direction of the suspension flow and magnetic field. That’s why, the motion of the suspension decreases due to the Hall parameter *m* upto $\zeta = 0.50$ and then increases as shown in Fig. 2. The resistive type forces are related to magnetic field hence as the magnetic field parameter *M* increases on the values 1.00, 2.00, 3.00 and 4.00, the radial velocity $\hbox {f}^{\prime }$($\zeta$) increases for a moment and then decreases from $\zeta = 0.60$ which is evident from Fig. 3. The present system is strongly dependent on rotation so if the rotation parameter $\Omega$ is increased for different positive values, then the radial velocity is reduced at the beginning and then enhanced as shown in Fig. 4. Figure 5 shows that at lower disk, the flow is decreased while at upper disk the motion is enhanced due to Reynolds number *Re*. Figure 6 shows that the radial velocity increaes till $\zeta = 0.40$ and then opposite effect is shown as the stretching parameter $\hbox {k}_{2}$ increases. Figure 7 reveals that as the stretching parameter $\hbox {k}_{3}$ due to upper disk is increased, the radial veocity $\hbox {f}^{\prime }$($\zeta$) is decreased upto $\zeta = 0.70$ and then increases.

**Figure 8:**
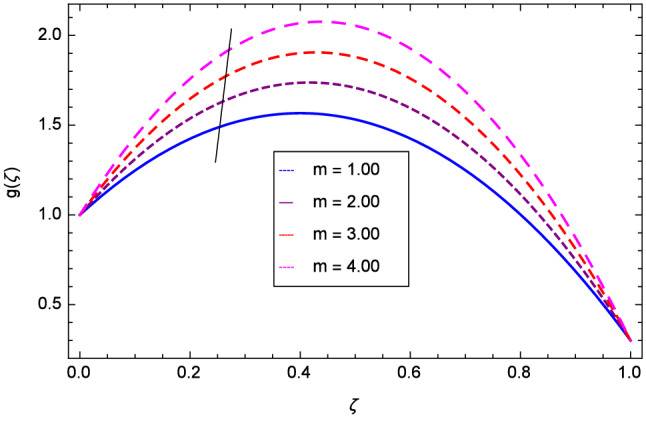
Analysis through Hall parameter *m* and tangential velocity *g*($\zeta$).

**Figure 9:**
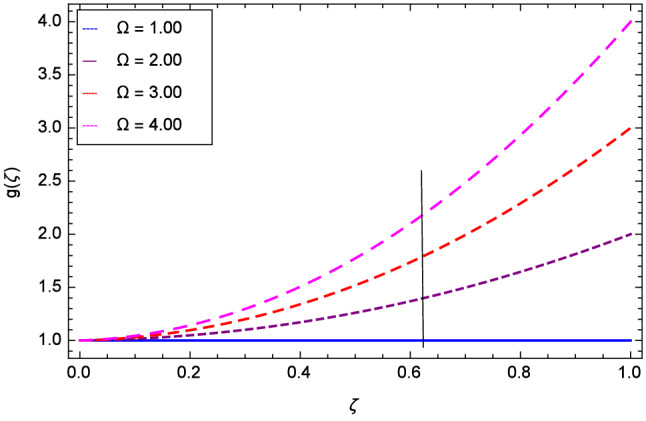
Analysis through rotation parameter $\Omega$ and tangential velocity *g*($\zeta$).

**Figure 10:**
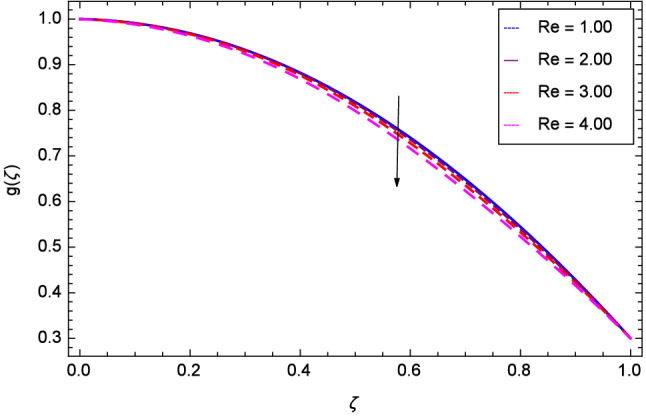
Analysis through Reynolds number *Re* and tangential velocity *g*($\zeta$).

**Figure 11:**
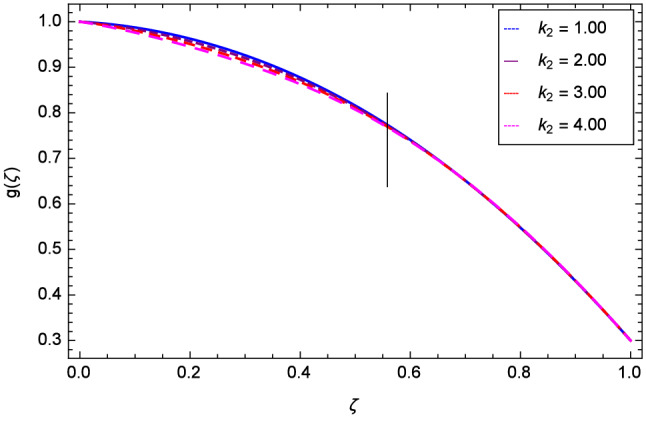
Analysis through lower disk stretching parameter $k_{2}$ and tangential velocity *g*($\zeta$).

**Figure 12:**
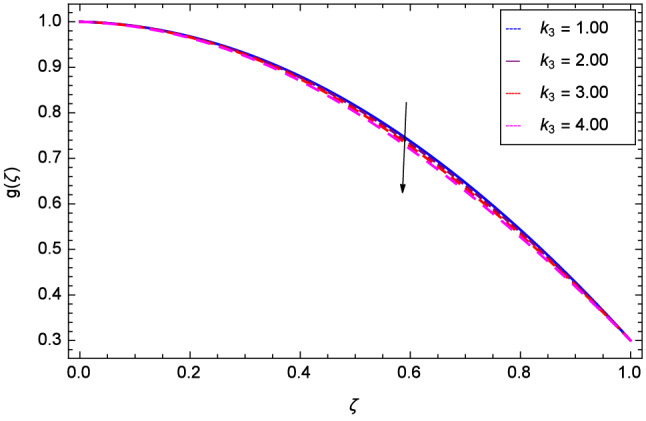
Analysis through upper disk stretching parameter $k_{3}$ and tangential velocity *g*($\zeta$).

The effect of Hall parameter *m* on tangential velocity *g*($\zeta$) is shown through Fig. 8. It is observed that the velocity is increased as the Hall parameter *m* assumes the values 1.00, 2.00, 3.00 and 4.00. Similarly increasing behavior is shown in Fig. 9 for tangential velocity as the rotation parameter have positive values. The reason is that increasing rotation parameter results in intensifying the centrifugal force which creates pressure on the suspension to enhance the motion sharply. Reynolds number is related to the thickness of the fluid so if this parameter is increased on the positive values, the tangential velocity *g*($\zeta$) is automatically reduced as depicted in Fig. 10. Figures 11 and 12 are related with stretching parameters $\hbox {k}_{2}$ and $\hbox {k}_{3}$ respectively. In both figures, the motion is reduced at lower and upper disks.

### Temperature profile

**Figure 13:**
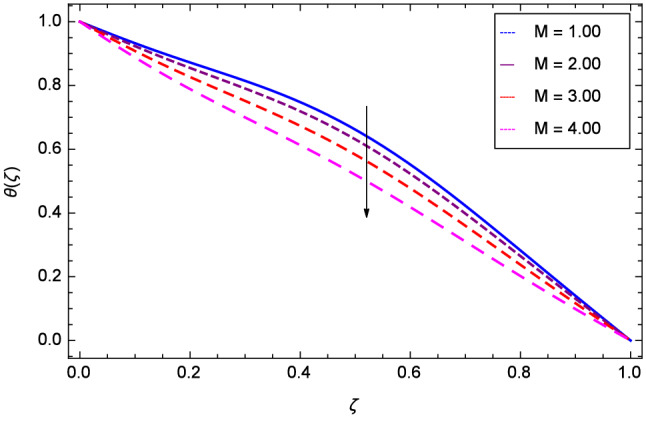
Analysis through magnetic field parameter *M* and temperature $\theta$($\zeta$).

**Figure 14:**
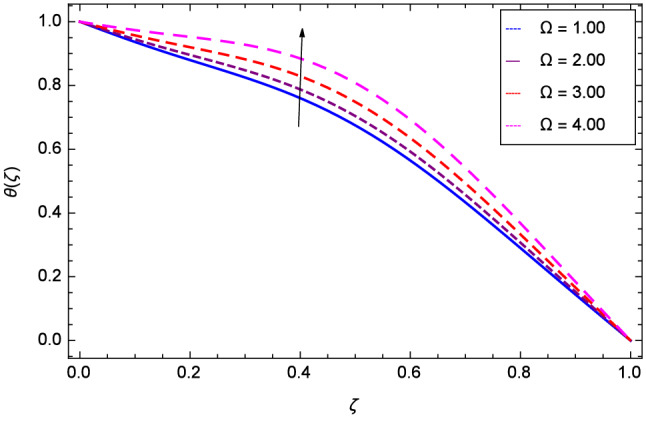
Analysis through rotation parameter $\Omega$ and temperature $\theta$($\zeta$).

**Figure 15:**
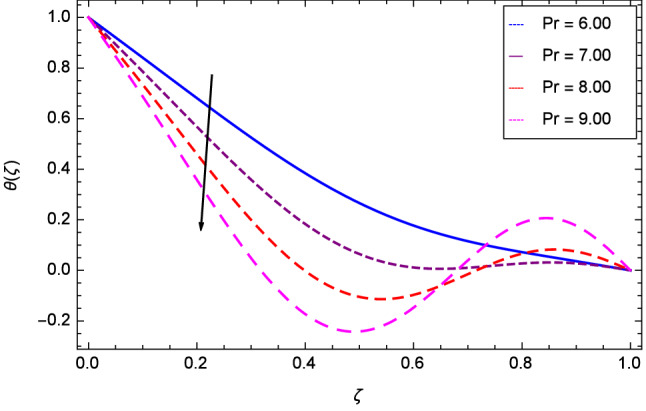
Analysis through Prandtl number *Pr* and temperature $\theta$($\zeta$).

**Figure 16:**
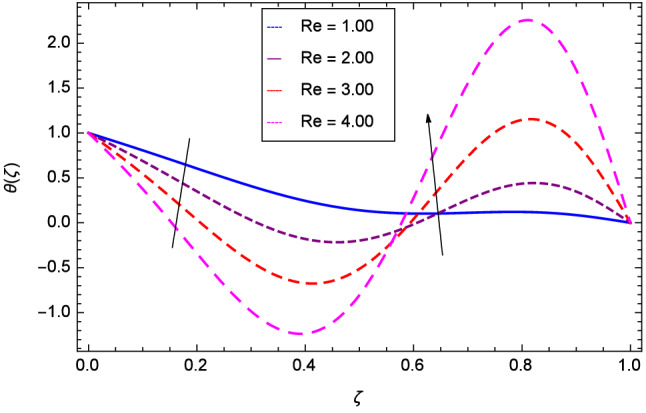
Analysis through Reynolds number *Re* and temperature $\theta$($\zeta$).

**Figure 17:**
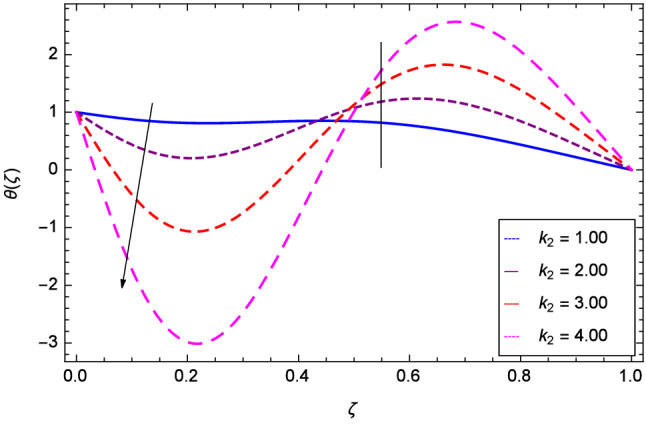
Analysis through stretching parameter $k_{2}$ at lower disk and temperature $\theta$($\zeta$).

**Figure 18:**
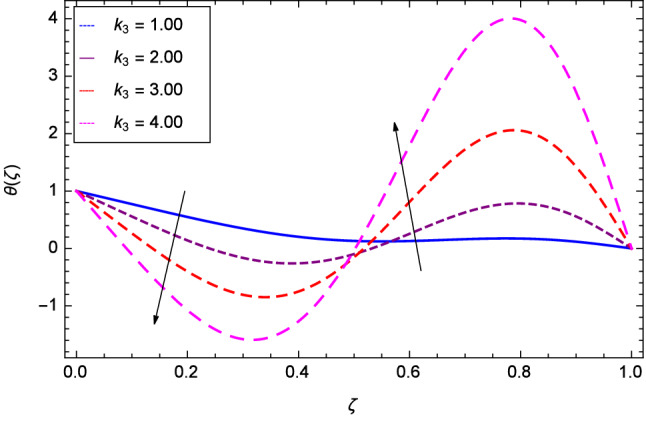
Analysis through stretching parameter $k_{3}$ at upper disk and temperature $\theta$($\zeta$).

Figure 13 projects that temperature falls down due to the increase in magnetic field parameter *M*. The rotation parameter $\Omega$ influence is represented in Fig. 14 where the temperature is enhanced by the dynamics of lower and upper disks. Cooling phenomena is obtained through the effect of Prandtl number *Pr* on temperature $\theta$($\zeta$). The larger values of *Pr* decrease the temperature as shown in Fig. 15. Similarly, Fig. 16 represents that temperature is dereased at lower disk and increased at upper disk when the Reynolds number is enlarged through the values 1.00, 2.00, 3.00 and 4.00. It has been observed in Figs. 17 and 18 that as the stretching parameters $k_{2}$, $k_{3}$ increase, the temperature is decreased at lower disk and increased at upper disk.

### Nanoparticles concentration profile

**Figure 19:**
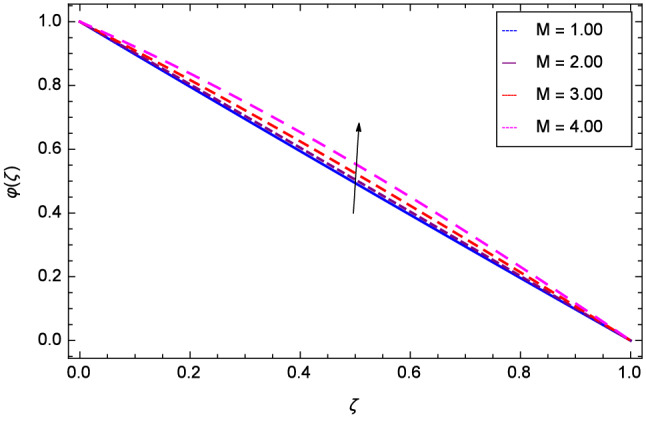
Analysis through magnetic field parameter *M* and nanoparticles concentration $\varphi$($\zeta$).

**Figure 20:**
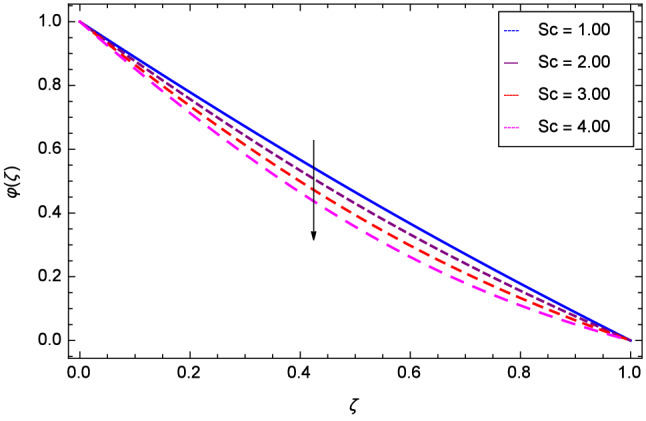
Analysis through Schmidt number *Sc* and nanoparticles concentration $\varphi$($\zeta$).

**Figure 21:**
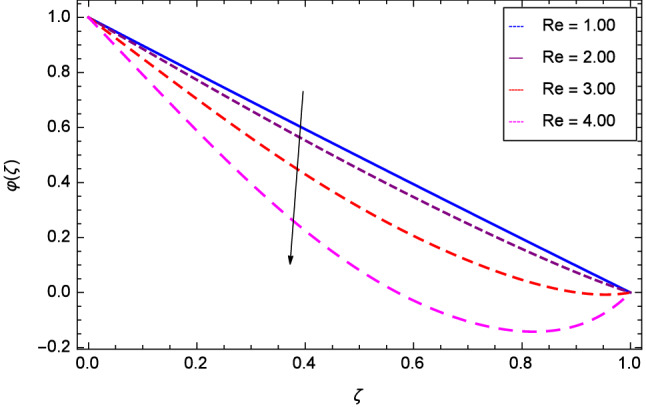
Analysis through Reynods number *Re* and nanoparticles concentration $\varphi$($\zeta$).

**Figure 22:**
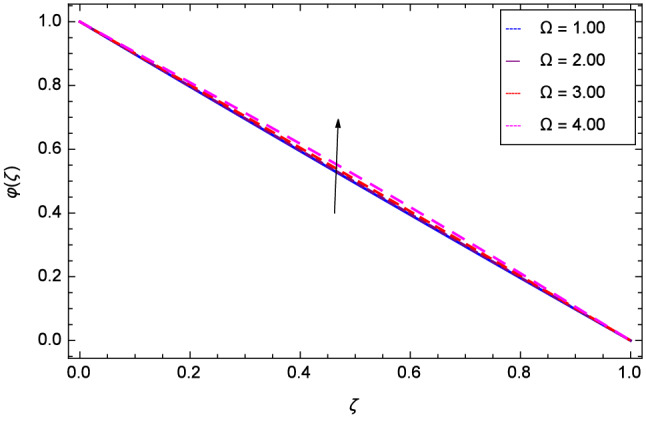
Analysis through rotation parameter $\Omega$ and nanoparticles concentration $\varphi$($\zeta$).

**Figure 23:**
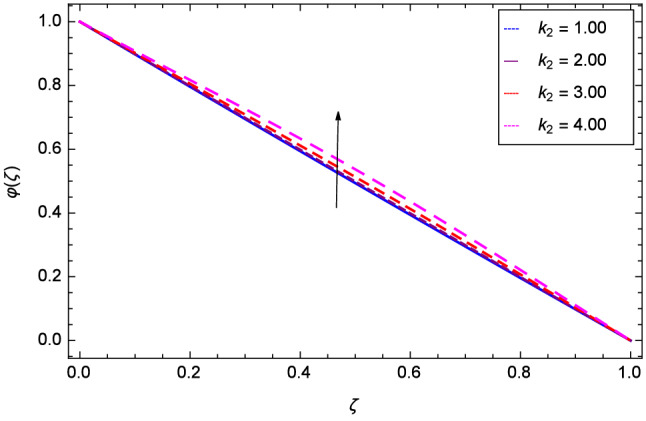
Analysis through stretching parameter $k_{2}$ at lower disk and nanoparticles concentration $\varphi$($\zeta$).

**Figure 24:**
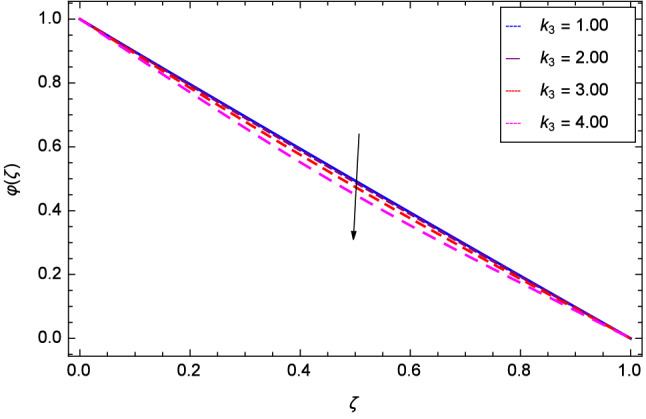
Analysis through stretching parameter $k_{3}$ at upper disk and nanoparticles concentration $\varphi$($\zeta$).

**Figure 25:**
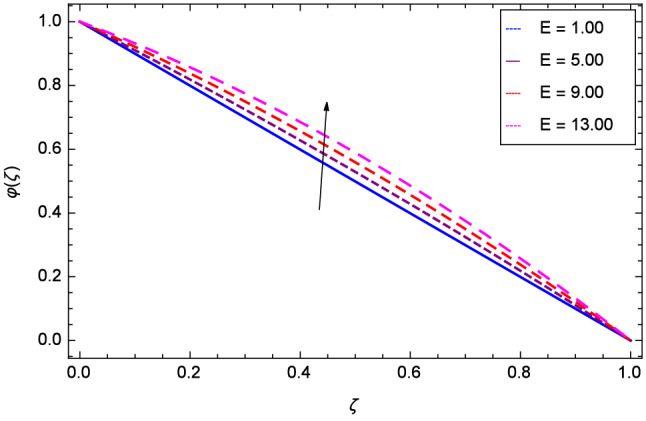
Analysis through Arrhenius activation energy parameter *E* and nanoparticles concentration $\varphi$($\zeta$).

**Figure 26:**
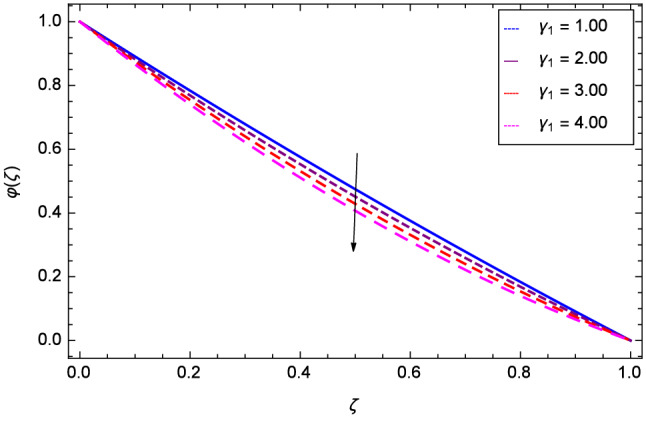
Analysis through chemical reaction parameter $\gamma _{1}$ and nanoparticles concentration $\varphi$($\zeta$).

**Figure 27:**
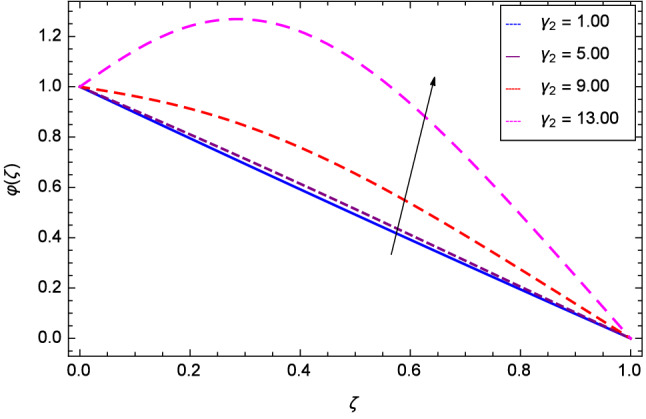
Analysis through temperature difference parameter $\gamma _{2}$ and nanoparticles concentration $\varphi$($\zeta$).

Figure 19 considers the nanoparticles concentration profile $\varphi$($\zeta$) and magnetic field parameter *M*. It is observed that the nanoparticle concentration is boost up with the high estimation of magnetic field strength. Lorentz forces due to magnetic field expedite the nanoparticle concentration. The influence of Schmidt number *Sc* is depicted in Fig. 20. Due to Schmidt number *Sc*, the nanofluids shrink and so decrease the concentration. Physically, it shows that nanoparticles addition declines the viscosity of a conventional regular fluid, i.e. water in the present case. Figure 21 explains that Reynolds number *Re* decreases the nanoparticles concentration $\varphi$($\zeta$). Physically, the Reynolds number is related to the motion of the fluid. Since nanoparticles are involved in the present system so Reynolds number has decreasing effect on nanoparticles concentration. Figure 22 demonstrates that as the rotation parameter $\Omega$ increases, the nanoparticles concentration is enhanced. Physically, hybrid nanofluid suspension increases the thermal energy which consequently enhances the nanofluid concentration. Figure 23 is sketched for the role of stretching parameter $k_{2}$ due to lower disk and nanoparticles concentration profile $\varphi$($\zeta$). Fluid and nanoparticles converge to the lower portion of the system i.e. lower disk hence the nanoparticles concentration is high. The reason is that stretching and tendency of fluid result in upserging the concentration. Figure 24 suggests that nanoparticles concentration profile $\varphi$($\zeta$) tends to decreasing on increasing the stretching parameter $k_{3}$ due to upper disk. Physically at high place, the fluid is found less in amount compared to the lower portion in the current dynamical systems so nanoparticles concentration profile $\varphi$($\zeta$) is weak. Figure 25 is used to portray the efficiency of Arrhenius activation energy. It depicts that nanoparticles concentration is developed with the high values of Arrhenius activation energy *E*. Figure 26 presents that on account of increasing the binary chemical reaction $\gamma _{1}$, nanoparticles concentration profile $\varphi$($\zeta$) is fall down. Chemical reaction consumes the nanoparticles concentration on this occasion. Figure 27 focuses on the temperature difference parameter $\gamma _{2}$ and nanoparticles concentration profile $\varphi$($\zeta$). It discloses that nanoparticles concentration goes to maximum on the increasing values of temperature difference parameter $\gamma _{2}$.

## Conclusions

The rotating system with hybrid nanofluid flow is investigated for Hall current effect, Arrhenius activation energy with binary chemical reaction using the solution of optimal homotopy analysis method (OHAM). The base fluid is taken as water and the two types of nanoparticles are silicon dioxide and molybedenum disulfide. OHAM solution is used to show the investigations through the effects of all embedded parameters on different profiles in the sketched graphs. The conclusion is given as:

1. Both increasing and decreasing behaviors are shown by the radial velocity $f^{\prime }$($\zeta$) for the Hall, magnetic field, rotation, stretching parameters and Reynolds number.
2. Tangential velocity *g*($\zeta$) increases with Hall and rotation parameters while it decreases with Reynolds number and stretching parameters.
3. Heat transfer increases with rotation parameter and decreases with magnetic field parameter, Prandtl number while it has both increasing and decreasing behaviors for the Reynolds number and stretching parameters.
4. Nanoparticles concentration $\varphi$($\zeta$) increases with magnetic field, rotation and stretching due to lower disk, Arrhenius activation energy and temperature difference parameters while it decreases with Schmidt and Reynolds numbers, stretching due to upper disk and chemical reaction parameters.
5. The solution validation presents a nice agreement between the current and published work.

## Data Availability

Availability exists for whole of the data.

## List of symbols

*m*: Hall parameter
(*u*, *v*, *w*): Velocity components
(*r*, $$\vartheta $$, *z*): Cylindrical coordinates
$a_{1}$: Stretching rate at lower disk
$a_{2}$: Stretching rate at upper disk
*H*: Distance between disks
*Sc*: Schmidt number
$$k_{r}^{2}$$: Chemical reaction
*M*: Magnetic field parameter
$m_{1}$: Fitted rate constant
*Pr*: Prandtl number
$$E_{a}$$: Activation energy
*E*: Non-dimensional activation energy parameter
$$E_{i}$$, $$i = 1$$, 2, 3..., 10: Arbitrary constants
*Re*: Reynolds number
$$k_{nf}$$: Thermal diffusivity
$m_{2}$, $m_{3}$: Masses of the silicon dioxide and molybedenum disulfide nanoparticles
$T_{1}$, $C_{1}$: Temperature and concentration at lower disk
$T_{2}$, $C_{2}$: Temperature and concentration at upper disk
*Rd*: Thermal radiation parameter
$m_{f}$: Mass of the base fluid
$k_{2}$: Dimensionless stretching parameter due to lower disk
$k_{3}$: Dimensionless stretching parameter due to upper disk
*T*: Temperature
*P*: Pressure
$c_{p}$: Specific heat at constant pressure
$$D_{B}$$: Diffusivity
*B*: Chemical species
$f^{\prime }$($\zeta$): Dimensionless radial velocity
*g*($\zeta$): Dimensionless tangential velocity
$B_{0}$: Applied magnetic field strength

## Greek symbols

$$\Omega $$: Rotation parameter
$\Omega _{1}$, $\Omega _{2}$: Angular velocities at lower and upper disks
$$\sigma $$: Electrical conductivity
$$\epsilon $$: Pressure parameter
$\sigma _{1}$: Stefan–Boltzmann constant
$\psi$: Physical stream function
$$\zeta $$: Similarity variable
$$\varphi $$: Nanoparticles concentration
$\phi$: Total volume fraction of nanoparticles
$\phi _{1}$: Volume fraction of silicon dioxide
$\phi _{2}$: Volume fraction of molybedenum disulfide
$B_{i}$, $i = 1$, 2, 3, 4, 5: Dimensionless hybrid nanofluid constants
$$\theta $$($$\zeta $$): Dimensionless temperature
$\gamma _{1}$: Chemical reaction parameter
$\gamma _{2}$: Temperature difference parameter
$$\nu $$: Kinematic viscosity
$\mu$: Coefficient of viscosity
$$\rho $$: Density

## Subscripts

f: Base fluid
hnf: Hybrid nanofluid
s: Solid nanoparticle

## Superscripts

${\prime }$: Differentiation with respect to $\zeta$

## Competing ***Interests

No such interests exist.

## References

[1] Ali B, Naqvi RA, Mariam A, Ali L, Aldossary OM (2021). Finite element method for magnetohydrodynamic (MHD) tangent hyperbolic nanofluid flow over a faster/slower stretching wedge with activation energy. Mathematics.

[2] Hayat T, Rashid M, Imtiaz M, Alsaedi A (2017). MHD convective flow due to a curved surface with thermal radiation and chemical reactions. J. Mol. Liq..

[3] Bibi A, Xu H (2020). Peristaltic channel flow and heat transfer of Carreau magneto hybrid nanofluid in the presence of homogeneous/heterogeneous reactions. Sci. Rep..

[4] Sambath P, Pullepu B, Hussain T, Shehzad SA (2018). Radiated chemical reaction impacts on natural convective MHD mass transfer flow induced by a vertical cone. Result Phys..

[5] Sohail M, Ali U, Al-Mdallal Q, Thounthong P, Sherif EM, Alrabaiah H, Abdelmalek Z (2020). Theoretical and numerical investigation of entropy for the variable thermophysical characteristics of Couple stress material: Applications to optimization. Alex. Eng. J..

[6] Abdelmalek Z, Al-Khaled K, Waqas H, Aldabesh A, Khan SU, Musmar SA, Tlili I (2020). Bioconvection in Cross nano-materials with magnetic dipole impacted by activation energy, thermal radiation, and second order slip. Symmetry.

[7] Ramzan M, Bilal M, Chung JD (2017). Influence of homogeneous–heterogeneous reactions on MHD 3D Maxwell fluid with Cattaneo–Christov heat flux and convective boundary condition. J. Mol. Liq..

[8] Khan NS (2019). Entropy generation in MHD mixed convection non-Newtonian second-grade nanoliquid thin film flow through a porous medium with chemical reaction and stratification. Entropy.

[9] Khan NS, Zuhra S, Shah Q (2019). Entropy generation in two phase model for simulating flow and heat transfer of carbon nanotubes between rotating stretchable disks with cubic autocatalysis chemical reaction. Appl. Nanosci..

[10] Khan NS (2019). Hall current and thermophoresis effects on magnetohydrodynamic mixed convective heat and mass transfer thin film flow. J. Phys. Commun..

[11] Usman AH (2021). Development of dynamic model and analytical analysis for the diffusion of different species in non-Newtonian nanofluid swirling flow. Front. Phys..

[12] Lund LA, Omar Z, Khan I, Baleanu D, Nisar KS (2020). Convective effect on magnetohydrodynamic (MHD) stagnation point flow of Casson fluid over a vertical exponentially stretching/shrinking surface: Triple solutions. Symmetry.

[13] Siddiqui MA, Riaz A, Khan I, Nisar KS (2020). Augmentation of mixed convection heat transfer in a lid-assisted square enclosure utilizing micropolar fluid under magnetic environment: A numerical approach. Result Phys..

[14] Islam S, Zubair M, Tassaddiq A, Shah Z, Alrabaiah H, Kumam P, Khan W (2020). Unsteady ferrofluid slip flow in the presence of magnetic dipole with convective boundary conditions. IEE. Access..

[15] Beg OA, Zueco J, Lopez-ochoa LM (2011). Network numerical analysis of optically thick hydromagnetic slip flow from a porous spinning disk with radiation flux, variable thermophysical properties, and surface injections effects. Chem. Eng. Commun..

[16] Agrawal P, Dadheech PK, Jat RN, Bohra M, Nisar KS, Khan I (2020). Lie similarity analysis of MHD flow past a stretching surface embedded in porous medium along with imposed heat source/sink and variable viscosity. J. Mater. Res. Technol..

[17] El-Kabeir SMM, El-Hakiem MA, Rashad AM (2008). Group method analysis of combined heat and mass transfer by MHD non-Darcy non-Newtonian natural convection adjacent to horizontal cylinder in a saturated porous medium. Appl. Math. Model..

[18] Alqahtani AM, Adnan, Khan U, Ahmed N, Mohyud-Din ST, Khan I (2020). Numerical investigation of heat and mass transport in the flow over a magnetized wedge by incorporating the effects of cross-diffusion gradients: Applications in multiple engineering systems. Math. Prob. Eng..

[19] Khan NS, Gul T, Islam S, Khan W (2017). Thermophoresis and thermal radiation with heat and mass transfer in a magnetohydrodynamic thin film second-grade fluid of variable properties past a stretching sheet. Eur. Phys. J. Plus.

[20] Khan NS (2019). Influence of inclined magnetic field on Carreau nanoliquid thin film flow and heat transfer with graphene nanoparticles. Energies.

[21] Khan NS (2019). Study of two dimensional boundary layer flow of a thin film second grade fluid with variable thermo-physical properties in three dimensions space. Filomat.

[22] Khan NS, Zuhra S (2019). Boundary layer unsteady flow and heat transfer in a second grade thin film nanoliquid embedded with graphene nanoparticles past a stretching sheet. Adv. Mech. Eng..

[23] Khan M, Ali W, Ahmed J (2020). A hybrid approach to study the influence of Hall current in radiative nanofluid flow over a rotating disk. Appl. Nanosci..

[24] Sing JK, Begum SG, Seth GS (2018). Influence of hall current and wall conductivity on hydromagnetic mixed convective flow in a rotating Darcian channel. Phys. Fluids.

[25] Abdel-Wahed M, Akl M (2016). Effect of Hall current on MHD flow of a nanofluid with variable properties due to a rotating disk with viscous dissipation and nonlinear thermal radiation. AIP Adv..

[26] Gosh SK, Beg OA, Narahari M (2009). Hall effects on MHD flow in a rotating system with heat transfer charateristics. Mecannica.

[27] Bilal M, Ramzan M (2019). Hall current effect on unsteady rotational flow of carbon nanotubes with dust particles and nonlinear thermal radiation in Darcy Forchheimer porous media. J. Therm. Anal. Calorim..

[28] Ahmad MW, McCash LB, Shah Z, Nawaz R (2020). Cattaneo–Christov heat flux model for second grade nanofluid flow with Hall effect through entropy generation over stretchable rotating disk. Coatings.

[29] Khan NS, Gul T, Islam S, Khan A, Shah Z (2017). Brownian motion and thermophoresis effects on MHD mixed convective thin film second-grade nanofluid flow with Hall effect and heat transfer past a stretching sheet. J. Nanofluids.

[30] Khan NS (2020). Lorentz forces effects on the interactions of nanoparticles in emerging mechanisms with innovative approach. Symmetry.

[31] Khan NS, Kumam P, Thounthong P (2021). Magnetic field promoted irreversible process of water based nanocomposites with heat and mass transfer flow. Sci. Rep..

[32] Ali F, Ahmad Z, Arif M, Khan I, Nisar KS (2020). A time fractional model of generalized Couette flow of Couple stress nanofluid with heat and mass transfer: Applications in engin oil. IEEE Access..

[33] Imtiaz A, Foong O, Aamina A, Khan N, Ali F, Khan I (2020). Generalized model of blood flow in a vertictical tube with suspension of gold nanomaterials: Applications in the cancer therapy. Comput. Mater. Contin..

[34] Kotnurkar AS, Katagi DC (2020). Bioconvective peristaltic flow of a third-grade nanofluid embodying gyrotactic microorganisms in the presence of Cu-blood nanoparticles with permeable walls. Multidiscip. Model. Mater. Struct..

[35] Hayat T, Haider F, Muhammad T, Alsaedi A (2018). Numerical study for Darcy–Forchheimer flow of nanofluid due to an exponentially stretching curved surface. Result Phys..

[36] Akilu S, Baheta AT, Kadirgama K, Padmanabhan E, Sharma KV (2019). Viscosity, electrical and thermal conductivities of ethylene and propylene glycol-based $$\beta $$-SiC nanofluids. J. Mol. Liq..

[37] Chilambarasan L, Prakash R, Shanu JP, Murugasen P (2019). Investigation on the electrical conductivity of aqueous gycol based Zno nanofluids. J. Appl. Fluid Mech..

[38] Shehzad SA, Reddy MG, Rauf A, Abbas Z (2020). Bioconvection of Maxwell nanofluid under the influence of double diffusive Cattaneo–Christov theories over isolated disk. Phys. Scr..

[39] Waqas H, Khan SU, Hassan M, Bhatti MM, Imran M (2019). Analysis on the bioconvection flow of modified second-grade nanofluid containing gyrotactic microorganisms and nanoparticles. J. Mol. Liq..

[40] Khan NS (2017). Magnetohydrodynamic nanoliquid thin film sprayed on a stretching cylinder with heat transfer. Appl. Sci..

[41] Khan NS, Kumam P, Thounthong P (2020). Second law analysis with effects of Arrhenius activation energy and binary chemical reaction on nanofluid flow. Sci. Rep..

[42] Khan NS (2019). Slip flow of Eyring–Powell nanoliquid film containing graphene nanoparticles. A.I.P. Adv..

[43] Maraj EN, Iqbal Z, Azhar E, Mehmood Z (2018). A comprehensive shape factor analysis using transportation of $$\text{MoS}_{2}$$-$$\text{ SiO}_{2}/\text{H}_{2}\text{ O }$$ inside an isothermal semi vertical inverted cone with porous boundary. Result Phys..

[44] Salehi S, Nori A, Hosseinzadeh Kh, Ganji DD (2020). Hydrothermal analysis of MHD squeezing mixture fluid suspended by hybrid nanoparticles between two parallel plates. Case Stud. Therm. Eng..

[45] Shah Z, Sheikholeslami M, Kumam P, Ullah I, Shafee A (2020). Modeling of entropy optimization for hybrid nanofluid MHD flow through a porous annulus involving variation of Bejan number. Sci. Rep..

[46] Khan MI, Khan SA, Hayat T, Waqas M, Alsaedi A (2020). Modeling and numerical simulation for flow of hybrid nanofluid ($$\text{ SiO}_{2}/\text{C}_{3}\text{ H}_{8}\text{ O}_{2}$$) and ($$\text{ MoS}_{2}/\text{C}_{3}\text{ H}_{8}\text{ O}_{2}$$) with entropy optimization and variable viscosity. Int. J. Numer. Methods Heat Fluid Flow.

[47] Wahid NS, Arifin NM, Khashi’ie NS, Pop I (2021). Hybrid nanofluid slip flow over an exponentially stretching/shrinking permeable sheet with heat generation. Mathematics.

[48] Muhammad K, Hayat T, Alsaedi A, Ahmed B (2021). A comparative study for convective flow of basefluid (gasoline oil), nanomaterial (SWCNTs) and hybrid nanomaterial (SWCNTs + MWCNTs). Appl. Nanosci..

[49] Khan NS, Kumam P, Thounthong P (2019). Renewable energy technology for the sustainable development of thermal system with entropy measures. Int. J. Heat Mass Transf..

[50] Khan NS, Kumam P, Thounthong P (2020). Computational approach to dynamic systems through similarity measure and homotopy analysis method for the renewable energy. Crystals.

[51] Khan U, Shafiq A, Zaib A, Baleanu D (2020). Hybrid nanofluid on mixed convective radiative flow from an irregular variably thick moving surface with convex and concave effects. Case Stud. Therm. Eng..

[52] Khan NS, Shah Z, Shutaywi M, Kumam P, Thounthong P (2020). A comprehensive study to the assessment of Arrhenius activation energy and binary chemical reaction in swirling flow. Sci. Rep..

[53] Zuhra S, Khan NS, Islam S, Nawaz R (2020). Complexiton solutions for complex KdV equation by optimal homotopy asymptotic method. Filomat.

